# Effect of Reactive Oxygen Species Generation in Rabbit Corneal Epithelial Cells on Inflammatory and Apoptotic Signaling Pathways in the Presence of High Osmotic Pressure

**DOI:** 10.1371/journal.pone.0072900

**Published:** 2013-08-15

**Authors:** Yihui Chen, Min Li, Bing Li, Weifang Wang, Anjuan Lin, Minjie Sheng

**Affiliations:** Department of Ophthalmology, Shanghai Tenth People's Hospital, Tongji University, Shanghai, China; UAE University, Faculty of Medicine & Health Sciences, United Arab Emirates

## Abstract

It is generally accepted that high osmotic pressure (HOP) of lacrimal fluid is the core mechanism causing ocular inflammation and injury. However, the association between HOP and the regulation of cell inflammatory response and apoptotic pathways remains unclear. In the present study, we used HOP to interfere with in vitro cultured rabbit corneal epithelial cells, and found that HOP increased the generation of reactive oxygen species (ROS) in rabbit corneal epithelial cells, and increased ROS in turn induced the activation of JNK inflammatory signaling pathway, which further promoted the expression of pro-inflammatory factor NF-κβ and induced the generation of inflammatory factor IL-1β and TNF-α. In addition, HOP-induced ROS in rabbit corneal epithelial cells regulated the CD95/CD95L-mediated cell apoptotic signaling pathway by activating JNK inflammatory signaling pathway. These findings may serve as new theoretical basis and a new way of thinking about the treatment of ocular diseases, especially dry eye.

## Introduction

It is generally accepted that high osmotic pressure (HOP) of lacrimal fluid is the core link causing ocular inflammation and injury, and is closely associated with various ocular diseases and disorders such as dry eye, ocular inflammation, and ocular discomfort related to the wearing of contact lens [1,2]. Studies [3,4] have demonstrated that HOP promotes the release of inflammatory mediators into lacrimal fluid by activating inflammatory cascade, which causes inflammatory response and injury to corneal epithelial cells, resulting in tear membrane instability. This instability in turn exacerbates the ocular HOP, forming a positive feedback effect and finally causing the occurrence of corneal and other ocular surface diseases [5]. However, there is no convincing explanation about the association between HOP and the regulation of cell inflammatory response and the apoptotic signaling pathway.

Reactive oxygen species (ROS) are by-products of cellular metabolism and play important roles in regulating cell signaling pathways [6]. It was found that the content of ROS in corneal fibroblasts of keratoconus patients and corneal epithelial cells of dry eye animal models was elevated, suggesting that there may be a close pathological association between intracellular accumulation of ROS and corneal surface imbalance [7,8].

High osmotic pressure is a potential pro-inflammatory factor. Ample studies have demonstrated that the content of ROS is increased in different tissue cells such as cardiomyocytes and hepatocytes under HOP intervention, thus affecting the normal cell function [9,10]. However, there are few studies about ROS changes under HOP, and whether HOP participates in inflammatory response and mediates the regulation of cell apoptosis in ocular surface cells.

The aim of this study is to investigate whether HOP increases the generation of ROS in rabbit corneal epithelial cells (RCECs), and the increased ROS induce inflammatory and apoptotic signaling pathways. We examined that HOP increased the generation of ROS in RCECs, and the increased ROS in turn induced the activation of the JNK inflammatory signaling pathway and regulated the CD95/CD95L-mediated cell apoptotic signaling pathway.

## Results

### RCECs culture

**Figure 1:**
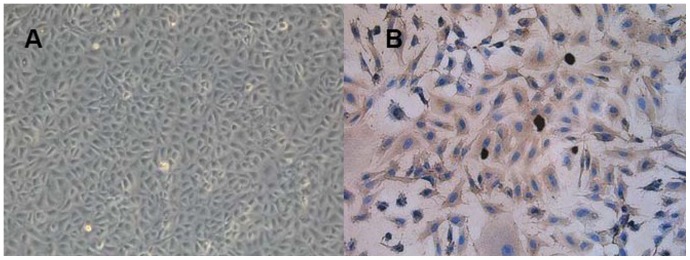
Culture and identification of RCECs under light microscopy. Pure RCECs were obtained at the second passage (A). RCECs were detected positive for cytokeratin3/2p (B). A: Original*100. B: Original*200.

RCECs grew well in the media and were spindle-shaped. Pure RCECs were obtained after 2–3 passages from the media (Fig. 1A). RCECs were positive for cytokeratin3/2p (Fig. 1B).

### Detection of ROS

**Figure 2:**
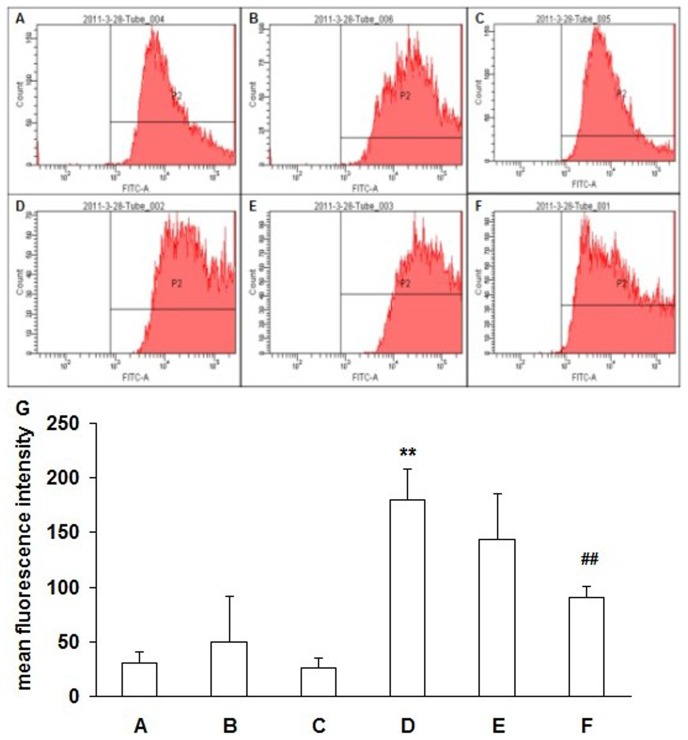
Detection of ROS by flow cytometry. RCECs were cultured in normal osmotic pressure(A), normal osmotic pressure added with NAC (B) or DPI (C), and 90 mM NaCl high osmotic pressure (HOP) group (D), 90 mM NaCl HOP added with NAC (E) or DPI (F). Intracellular ROS levels in RCECs were detected using DCFH-DA. Intracellular ROS levels increased in the HOP group. ROS levels decreases in the HOP added with NAC and DPI group. Statistics (G) showed that the fluorescence intensity in the 90 mM NaCl HOP group was significantly higher than that in the normal osmotic pressure group (*P*<0.01), and the fluorescence intensity in the HOP group added with ROS inhibitor NAC and DPI decreased to some extent. The decrease was statistically significant in the HOP group added with DPI(*P*<0.01). ** *P*<0.01 vs. A, ## *P*<0.01 vs. D.

Fluorescence expression of cells was detected by flow cytometry. Only the negative peak was detected in the blank control group under the FITC assay conditions (Fig. 2A). The fluorescence intensity of the HOP group was significantly higher than that of the normal osmotic pressure group, and the positive peak had a rightward deviation as shown by the displacement diagram. The intracellular fluorescence intensity in the HOP added with ROS inhibitor diphenyliodonium (NADPH oxidase inhibitor, DPI) (Sigma, St. Louis, MO, 10 µM), or N-Acetyl-L-cysteine (Antioxidant and free radical scavenger, NAC) (Sigma, St. Louis, MO, 10 mM) was attenuated as compared with that in the HOP group without addition of the ROS inhibitors, and the positive peak had a leftward deviation (Fig. 2B-F). Statistics showed that the fluorescence intensity in the 90 mmol/L NaCl HOP group was significantly higher than that in the normal osmotic pressure group (*P*<0.01), and the fluorescence intensity in the HOP group added with ROS inhibitor NAC and DPI decreased to some extent. The decrease was statistically significant in the HOP group added with DPI (*P*<0.01) (Fig. 2G).

### Protein expression of Jun N-terminal kinase (JNK)and phosphorylated-Jun N-terminal kinase (p-JNK)

**Figure 3:**
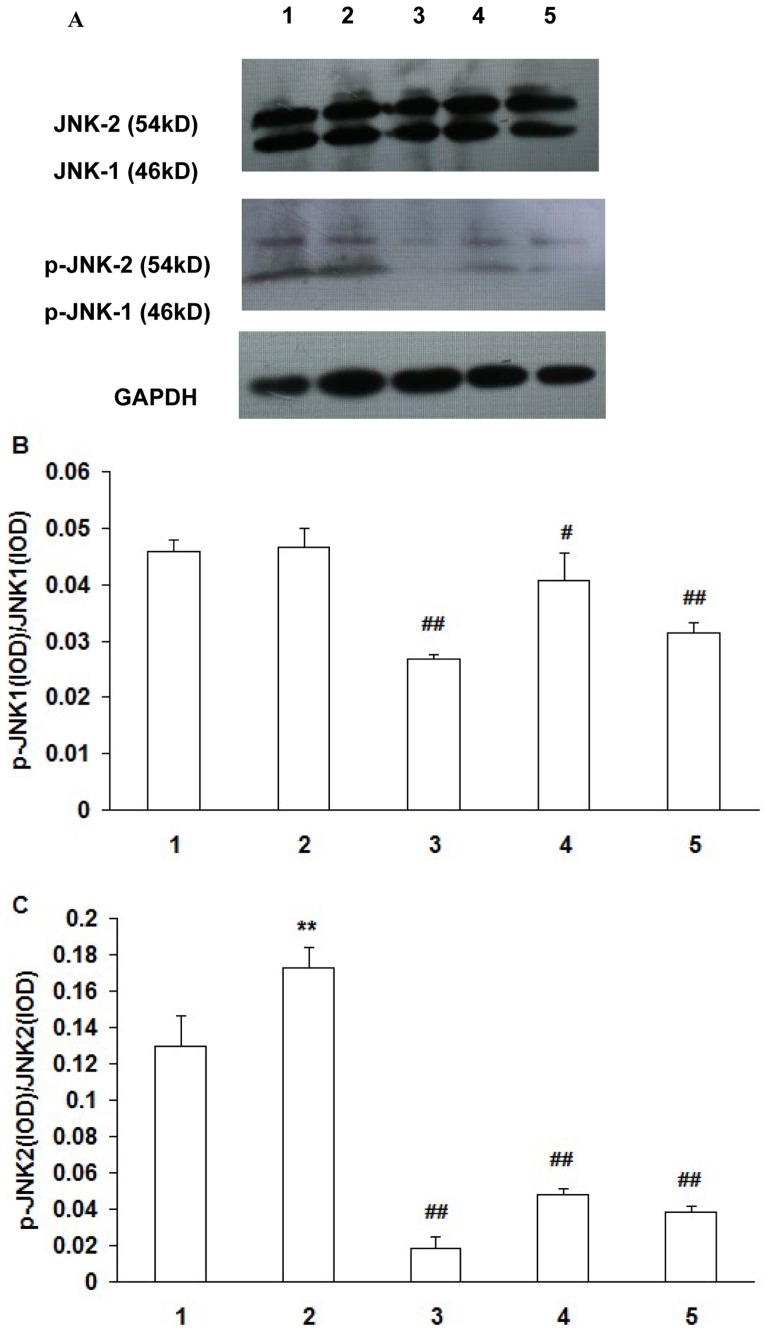
Expression of JNK1, JNK2, p-JNK1 and p-JNK2 protein in RCECs. RCECs cultured in NOP (normal osmotic pressure, 312mOsM) (1), HOP (high osmotic pressure, 500mOsM) (2), HOP+NAC (10 mM) (3), HOP+ DPI (10 µM) (4), HOP+ JNK inhibitor SP600125 (25 µM) (5) for 24 h. There was no significant difference in p-JNK-1/JNK-1 between the HOP group and the NOP group. Compared with the HOP group, p-JNK-1/JNK-1 levels decreased significantly in the HOP group added with ROS inhibitor NAC,DPI and JNK inhibitor SP600125. The p-JNK-2/JNK-2 ratio was increased in the HOP group compared with the NOP group. Compared with the HOP group, p-JNK-2/JNK-2 ratio was significantly decreased in HOP group added with ROS inhibitor NAC, DPI and JNK inhibitor SP600125. (mean±SD, n = 3) ** *P*<0.01 vs. NOP, # *P*<0.05 vs. HOP, ## *P*<0.01 vs. HOP.

There was no significant difference in p-JNK-1/JNK-1 between the HOP group and the normal osmotic pressure group (*P*>0.05). Compared with the HOP group without addition of the inhibitors, p-JNK-1/JNK-1 levels decreased significantly in the HOP group added with ROS inhibitor NAC and JNK inhibitor SP600125 (*P*<0.01), and in the HOP group added with ROS inhibitor DPI (*P*<0.05) (Fig. 3A,3B).

Compared with the normal osmotic pressure group, the p-JNK-2/JNK-2 ratio was increased in the HOP group (*P*<0.01). Compared with the HOP group without addition of the inhibitors, the p-JNK-2/JNK-2 ratio was significantly decreased in HOP group added with ROS inhibitor NAC, DPI and JNK inhibitor SP600125 (*P*<0.01) (Fig. 3A,3C).

### Detection of RCECs p-JNK expression by ELISA

**Figure 4:**
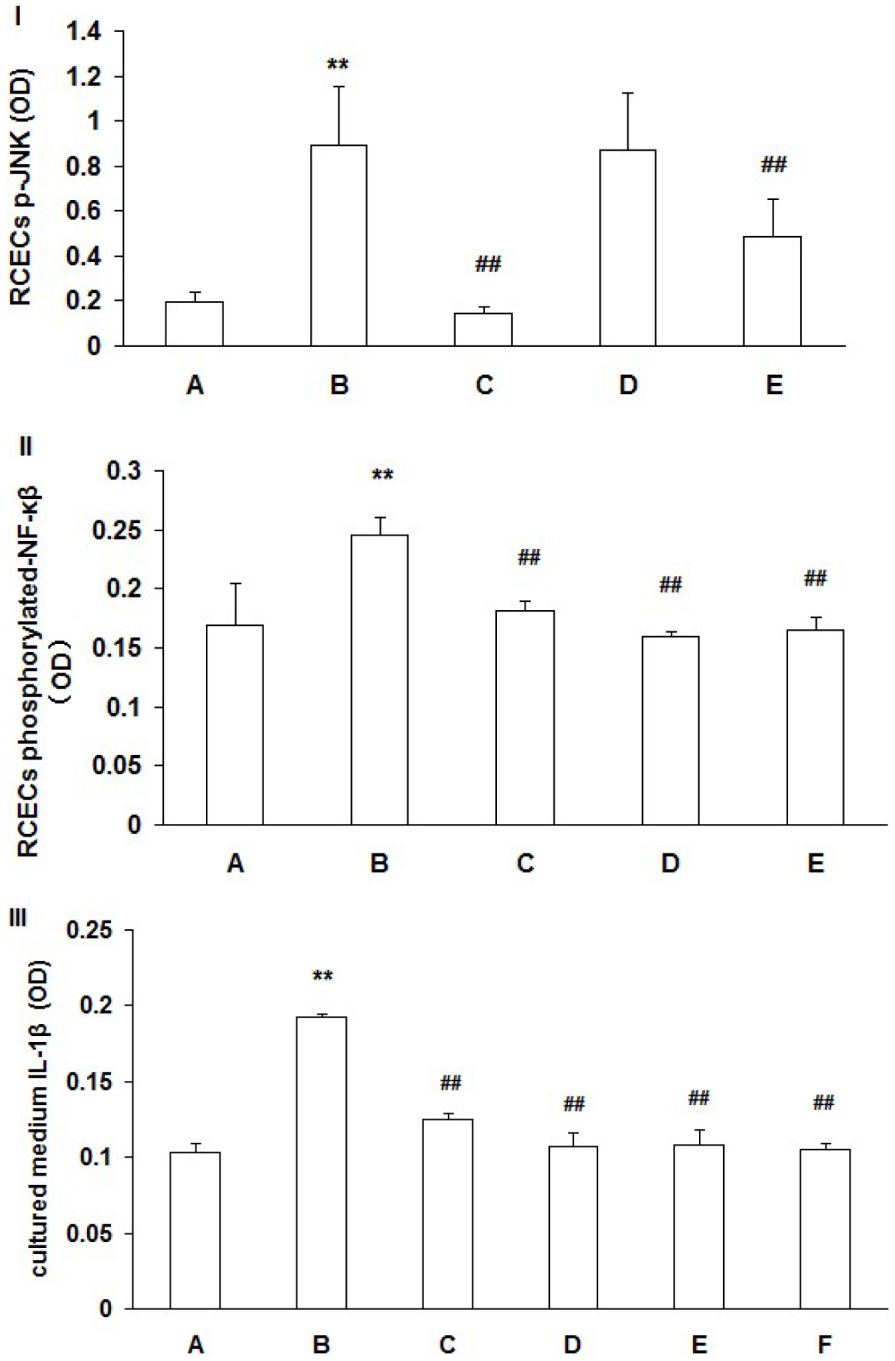
RCECs p-JNK (I), phosphorylated-NF-κβ (II) and cultured medium IL-1β(III) levels. RCECs cultured in NOP (normal osmotic pressure, 312mOsM) (A), HOP (high osmotic pressure, 500mOsM) (B), HOP+NAC (10 mM) (C), HOP+ DPI (10 µM) (D), HOP+ JNK inhibitor SP600125 (25 µM) (E) and HOP+ NF−kB inhibitor Bay11-7082(5 µM) (F) for 24 h. p-JNK expression in the HOP group was significantly higher than that in the NOP group (*P*<0.01). p-JNK expression in the HOP group added with ROS inhibitor NAC and JNK inhibitor SP600125 was decreased significantly (*P*<0.01) (I). phosphorylated-NF-κβ expression in the HOP group was significantly higher than that in the NOP group (*P*<0.01). phosphorylated-NF-κβ expression in the the HOP group added with ROS inhibitor NAC, DPI and JNK inhibitor SP600125 was decreased significantly (*P*<0.01) (II). IL-1β expression in the HOP group was significantly higher than that in NOP group (*P*<0.01). IL-1β expression in the HOP group added with ROS inhibitor NAC, DPI, JNK inhibitor SP600125 and NF-κB inhibitor Bay11-7082 was decreased (*P*<0.01) (III). (mean±SD, n = 3). ** *P*<0.01 vs. group A, ## *P*<0.01 vs. group B.

RCECs p-JNK expression in the 90 mM NaCl HOP group was significantly higher than that in the normal osmotic pressure group (*P*<0.01). p-JNK expression in the 90 mM NaCl HOP group added with ROS inhibitor NAC and JNK inhibitor SP600125 was decreased significantly (*P*<0.01) (Fig. 4-I).

### Detection of RCECs phosphorylated-NF-κβ expression by ELISA

RCECs phosphorylated-NF-κβ expression in the 90 mM NaCl HOP group was significantly higher than that in the normal osmotic pressure group (*P*<0.01). Phosphorylated-NF-κβ expression in the 90 mM NaCl HOP group added with ROS inhibitor NAC, DPI and JNK inhibitor SP600125 was decreased significantly (*P*<0.01) (Fig. 4-II).

### Detection of cultured medium IL-1β expression by ELISA

Cultured medium IL-1β level in the 90 mM NaCl HOP group was significantly higher than that in the normal osmotic pressure group (*P*<0.01). IL-1β level in the 90 mM NaCl HOP group added with ROS inhibitor NAC, DPI, JNK inhibitor SP600125 and NF-κB inhibitor Bay11-7082 was decreased (*P*<0.01) (Fig. 4-III).

### Detection of RCECs TNF-a expression by RT-PCR

**Figure 5:**
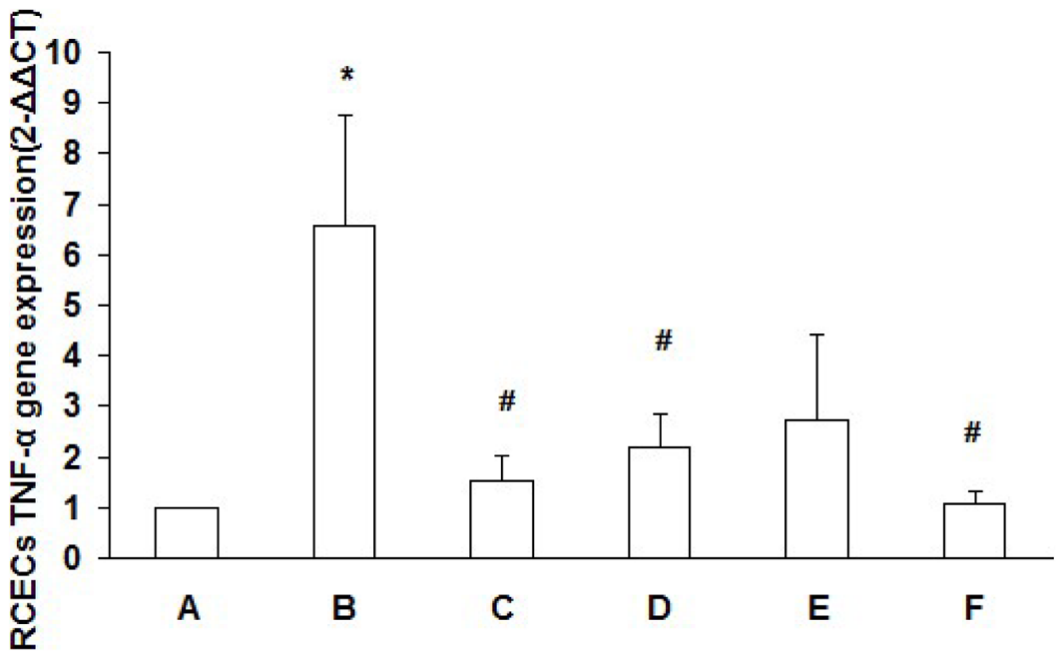
RCECs TNF-α gene expression. RCECs cultured in NOP (normal osmotic pressure, 312mOsM) (A), HOP (high osmotic pressure, 500mOsM) (B), HOP+NAC (10 mM) (C), HOP+ DPI (10 µM) (D), HOP+ JNK inhibitor SP600125 (25 µM) (E) and HOP+ NF−kB inhibitor Bay11-7082(5 µM) (F) for 24 h. RCECs TNF-a mRNA expression in the HOP group was significantly higher than that in the NOP group (*P*<0.05). TNF-a mRNA expression in the HOP group added with ROS inhibitor NAC, DPI and NF-kB inhibitor Bay11-7082 was decreased (*P*<0.05). (mean±SD, n = 3). * *P*<0.05 vs. group A, # *P*<0.05 vs. group B.

RCECs TNF-a mRNA expression in the 90mM NaCl HOP group was significantly higher than that in the normal osmotic pressure group (*P*<0.05). The TNF-a mRNA expression in the 90mM NaCl HOP group added with ROS inhibitor NAC, DPI and NF-kB inhibitor Bay11-7082 was decreased (*P*<0.05) (Fig. 5). The melting curve and amplification plots of the respective real-time PCR experiments were provided as Figure S1.

### CD95 and CD95L protein expression

**Figure 6:**
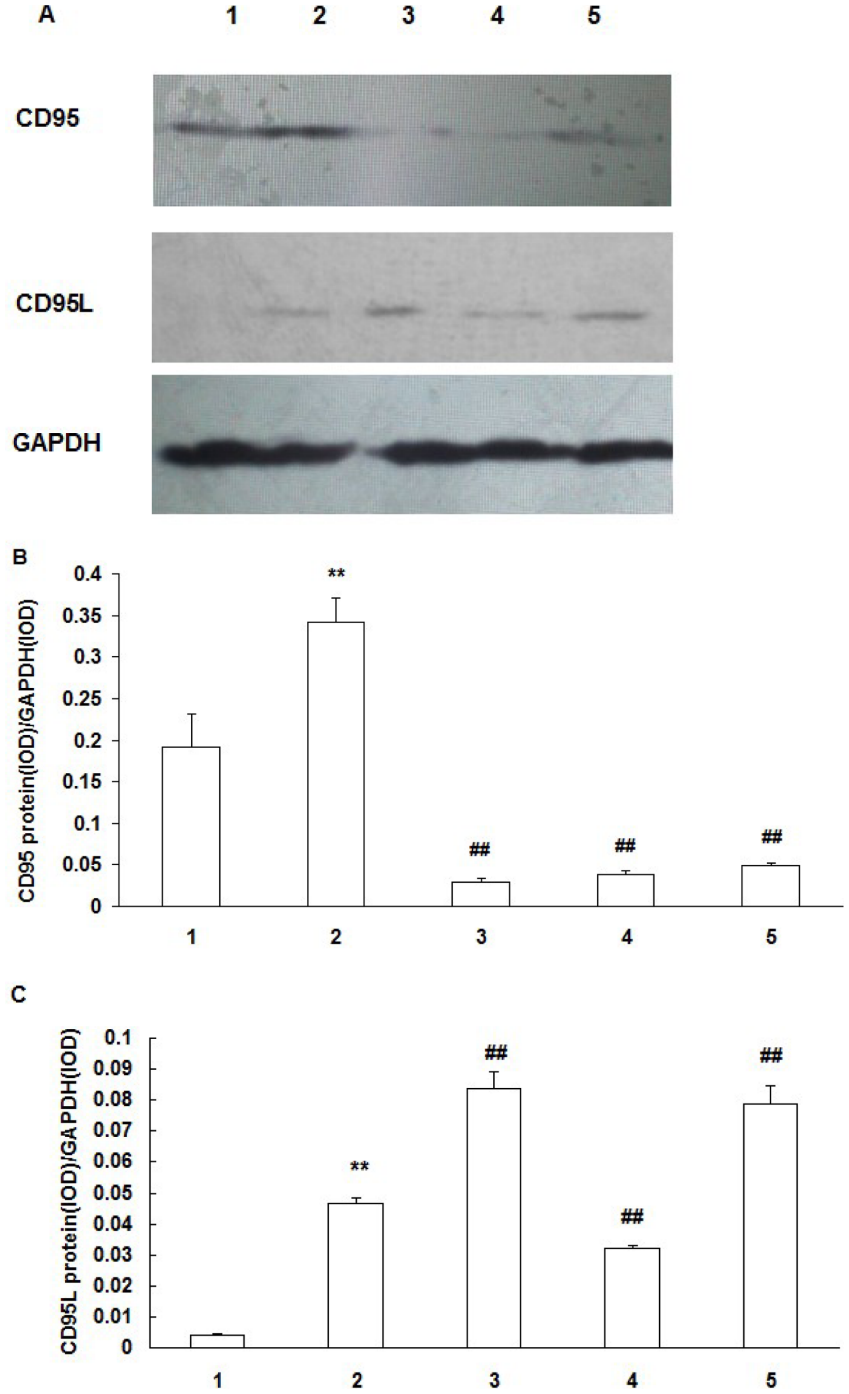
Expression of CD95 and CD95L protein in RCECs. A: western blotting analysis showing the presence of CD95 and CD95L protein in RCECs. B, C: Results of statistical analysis of protein levels relative to GAPDH. RCECs cultured in NOP (normal osmotic pressure, 312mOsM) (1), HOP (high osmotic pressure, 500mOsM) (2), HOP+NAC (10 mM) (3), HOP+ DPI (10 µM) (4), HOP+ JNK inhibitor SP600125 (25 µM) (5) for 24 h. CD95 and CD95L expression in the HOP group was significantly higher than that in NOP group (*P*<0.01). NAC, DPI and JNK inhibitor SP600125 downregulated CD95 expression in RCECs exposed to HOP. NAC and JNK inhibitor SP600125 upregulated CD95L expression in RCECs exposed to HOP, while CD95L expression was decreased significantly in the HOP group added with DPI (*P*<0.01). (mean±SD, n = 3). ** *P*<0.01 vs. NOP, ## *P*<0.01 vs. HOP.

RCECs CD95 expression in the HOP group was significantly higher than that in the normal osmotic pressure group (*P*<0.01). CD95 expression in the HOP group added with NAC, DPI and JNK inhibitor SP600125 was significantly decreased as compared with the HOP group without addition of the inhibitors (*P*<0.01) (Fig. 6A,B).

RCECs CD95L expression in the HOP group was significantly higher than that in the normal osmotic pressure group (*P*<0.01). CD95L expression in the HOP group added with NAC, JNK inhibitor SP600125 was significantly increased as compared with the HOP group (*P*<0.01), while CD95L expression was decreased significantly in the HOP group added with DPI (*P*<0.01) (Fig. 6A,C).

### Detection of cell apoptosis

**Figure 7:**
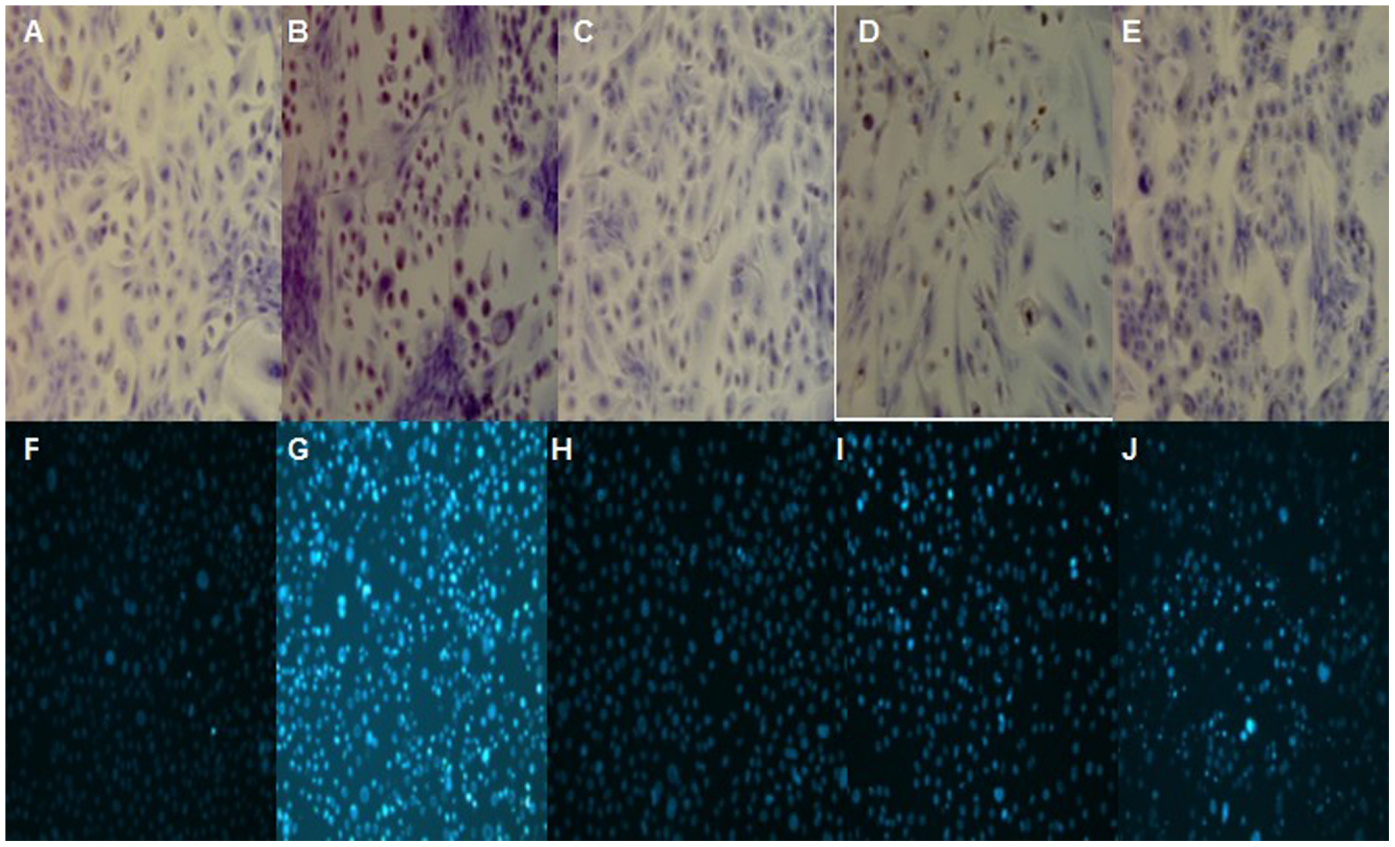
Detection of cell apoptosis. A-E: TUNEL assay; F-J: Hoechst33342 fluorescent staining assay. A and F: normal osmotic pressure group; B and G: 90 mM NaCl HOP group; C and H: 90 mM NaCl HOP added with NAC group; D and I: 90 mM NaCl HOP added with DPI group; E and J: 90 mM NaCl HOP added with SP600125 group. HOP increased RCECs apoptosis. NAC, DPI and SP600125 decreased cell apoptosis exposed to HOP.

Apoptosis of RCECs under HOP intervention was detected by TdT-mediated dUTP nick end labeling (TUNEL). It was found that about 80% RCECs in the 90 mM NaCl HOP group were positively stained; the nuclei were stained dark brown; and brownish yellow granules were seen in part of the cytoplasm. Most cells in the normal osmotic pressure group were negatively stained; only scattered nuclei were stained light yellow; and cell staining in the negative control group was negative. About 10% nuclei in the HOP group added with NAC were positively stained, while about 20% nuclei in the HOP group added with DPI and SP600125 were positively stained (Fig. 7A-E).

Fluorescent staining using Hoechst33342 kit showed that cells in the normal osmotic pressure group emitted low blue light; about 80% cells in the HOP group emitted high blue light; about 10% cells in the HOP group added with NAC emitted high blue light; about 20% cells in the HOP group added with DPI emitted high blue light; and about 40% cells in the HOP group added with SP600125 emitted high blue light (Fig. 7F-K).

**Figure 8:**
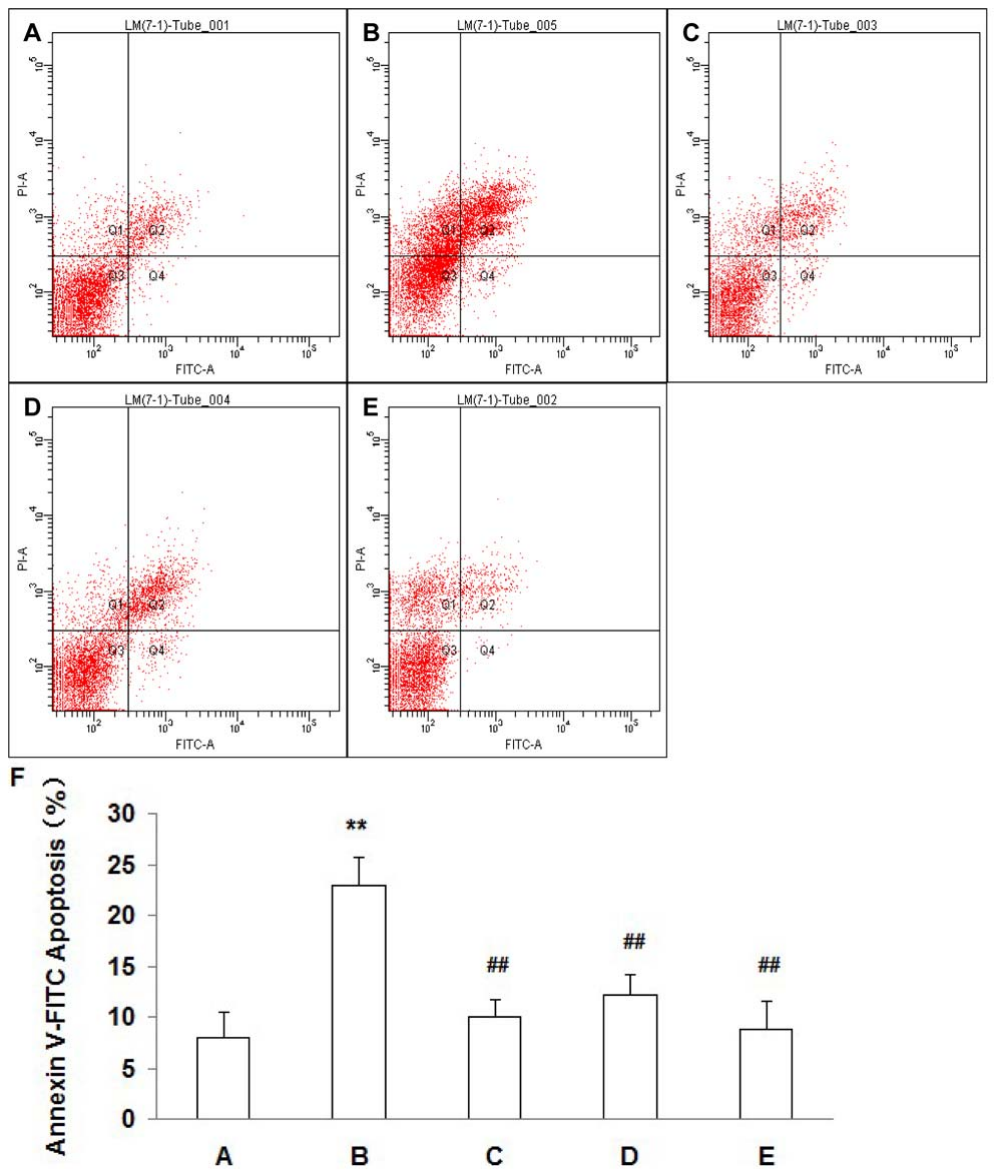
Detection of apoptotic cells with Annexin V assay. A: normal osmotic pressure group; B: 90 mM NaCl HOP group; C: 90 mM NaCl HOP added with NAC group; D: 90 mM NaCl HOP added with DPI group; E: 90 mM NaCl HOP added with SP600125 group. Statistics (F) showed that the percentage of apoptotic cells in the 90 mM NaCl HOP group was significantly higher than that in the normal osmotic pressure group (*P*<0.01), and the percentage of apoptotic cells in the HOP group added with NAC, DPI and SP600125 significantly decreased (*P*<0.01). (mean±SD, n = 3). ** *P*<0.01 vs. A, ## *P*<0.01 vs. B.

Apoptotic detected by flow cytometry showed that the percentage of apoptotic cells in the 90 mM NaCl HOP group was significantly higher than that in the normal osmotic pressure group (*P*<0.01), and the percentage of apoptotic cells in the HOP group added with NAC, DPI and SP600125 significantly decreased (*P*<0.01) (Fig. 8).

## Discussion

HOP is believed to be the core mechanism causing ocular surface pathological changes and injuries. Previous studies [11] have demonstrated that HOP could activate the JNK signaling pathway, promote NF-κB generation, and trigger the generation and expression of pro-inflammatory cytokine IL-1β,TNF-a and IL-8 in corneal epithelial cells, causing corneal epithelial injury. In addition, multiple studies [12–]14] have shown that HOP intervention on hepatocytes and cardiomyocytes would increase the production of ROS and promote the release of inflammatory factors, thus affecting the normal function of cells and causing the occurrence of some diseases. Oxidative stress could be a causative factor in the development of ocular surface disease, such as dry eye disease]15]. However, there are few studies reporting the production of ROS in ocular cells under HOP intervention and its mechanism in regulating the inflammatory signaling pathways and apoptosis.

It was found in our study that HOP intervention markedly increased the production of ROS in RCECs, and that NADPH oxidase inhibitor DPI and antioxidant NAC could reduce the production of ROS in RCECs induced by HOP. There was no significant difference in the degree of JNK-1 phosphorylation between the HOP group and the normal osmotic pressure group, indicating that HOP may not significantly affect JNK-1 activation, while p-JNK-1 expression in the HOP group added with NAC and DPI was reduced significantly as compared with the HOP group, indicating that inhibition of the ROS production in RCECs could reduce JNK-1 phosphorylation effectively. On the other hand, p-JNK-2 expression in the HOP group was significantly higher than that in the normal osmotic pressure group, indicating that HOP intervention on RCECs may be able to promote intracellular p-JNK-2 production, thus activating the JNK signaling pathway. In contrast, p-JNK-2 expression was reduced significantly in the HOP group added with NAC and DPI, indicating that reducing ROS production could effectively inhibit p-JNK-2 production. We therefore postulate that HOP intervention on RCECs could mainly promote p-JNK-2 production, and upstream ROS has a regulatory effect on the generation of p-JNK-1 and p-JNK-2.

NF-κB plays a key role in regulating transcription of mononuclear macrophage inflammatory response-related genes [16,17]. NF-κB could up-regulate gene transcription of TNF-a, IL-1β and other cytokines in inflammatory responses [18]. It was found in our study that IL-1β protein expression and TNF-a mRNA expression in the HOP group added with NF-κB selective inhibitor Bay11-7082 were decreased as compared with the HOP group, indicating that inhibition of NF-κB could reduce the production of IL-1β and TNF-a, and attenuate the injury to RCECs under the HOP condition. Our study also showed that the production of phosphorylated NF-κB was reduced in the HOP group added with selective inhibitor SP600125 of the JNK signaling pathway as compared with the 90 mmol/L NaCl HOP group, indicating that NF-κB production in RCECs was activated partly through the JNK signaling pathway.

Microenvironmental change of ocular epithelial cells would affect the normal physiological function of cells. HOP is a common ocular pathological environment and may induce abnormal increase in occular epithelial cell apoptosis [19]. Studies [20] have shown that increased ocular and lacrimal pro-apoptotic factors may activate apoptotic pathways. Interactions in apoptotic and inflammatory signaling pathways may cause ocular injuries.

CD95 (Fas) is a 48kDa single-chain transmembrane glycoprotein belonging to the tumor necrosis factor and nerve growth factor receptor superfamily. CD95L (FasL) is a ligand of CD95 and a type II membrane protein member of the TNF family. Both CD95 and CD95L are regulatory factors of cell apoptosis. Combination of CD95L in the form of a trimer with CD95 would cause trimerization of CD95, and subsequently the CD95 complex would enter cells via internalization, where it formed a death-inducing signaling complex or DISC, causing programmed cell death or apoptosis. Expression of the CD95/CD95L system in corneal tissues has an important impact on the normal physiological function and pathophysiology of corneal epithelial cells [21]. Mohan et al [22] demonstrated that the CD95/CD95L system could induce corneal epithelial apoptosis after corneal epithelial injury. Previous studies [10] found that ROS derived from NADPH oxidase under HOP intervention acted as an important up-stream signal and could induce CD95 tyrosine phosphorylation via the signaling pathway in which JNK and epithelial growth factor participated, which is the necessary condition for targeting-positioning of CD95 in the basal plasma membrane and inducing the formation of the apoptotic complex.

The eye is an immune privileged organ, with a relatively low corneal transplant rejection rate. Interaction in CD95 and CD95L is believed to be an important mechanism of maintaining the immune privilege status of the eye. CD95L is a natural immunosuppressant and can effectively prevent graft rejection [23,24]. Many studies [23–27] reported that the occurrence of rejection after corneal allografting was increased significantly in the absence of FasL. On the other hand, some other studies [28,29] demonstrated that the Fas/Fas-L system regulated immunoresponse by inducing cell apoptosis, and Fas-L expression could effectively inhibit inflammatory response. It was found in our study that the expression of CD95 and CD95L in RCECs was enhanced under HOP intervention, probably by activating the CD95/CD95L system and inducing apoptosis of the injured RCECs. As a result, HOP-induced injury to RCECs was attenuated. Antioxidant DPI effectively inhibited the production of CD95 and CD95L by inhibiting intracellular ROS, thus attenuating the activation of the CD95/CD95L-mediated apoptosis signaling pathway. It is indirectly evidenced that ROS, acting as an up-stream signal, could regulate CD95/CD95L-related apoptosis under the HOP condition. On the other hand, ROS inhibitor NAC had a different effect on CD95/CD95L. CD95L expression was increased after addition of NAC. Some studies demonstrated that some commonly used antioxidants had antioxidative and prooxidative dual actions. Sagristá et al [30] demonstrated that NAC could produce large amounts of ROS via autoxidation in an aerobic microenvironment. We postulate that NAC may not have the antioxidative role in some microenvironments; rather it may strengthen the intracellular oxidative stress, thus increasing the expression of CD95L.

HOP-induced ROS could activate epithelial growth factor receptor (EGFR) and JNK kinase, thus secondarily inducing CD95/EGFR separation and causing CD95 tyrosine phosphorylation [9]. As a result, the CD95 receptor underwent oligomeric reaction, causing CD95/EGFR protein complex to migrate to the basal plasma membrane, forming an apoptosis-inducing signaling complex and finally resulting in apoptosis of target liver cells. It was found in our study that CD95 expression was decreased and CD95L expression was increased in the HOP group added with JNK inhibitor SP600125 as compared with the HOP group without addition of the inhibitor. The difference was statistically significant. It is postulated that HOP may promote the generation of CD95 through the JNK signaling pathway, while the production of CD95L may be activated through some other signaling pathways. It may therefore conclude that inhibiting JNK activation would not affect CD95L production. The results obtained from TUNEL, Hoechst and flow cytometry methods showed that the positive staining rate in the HOP group added with JNK inhibitor SP600125 was lower than that in the HOP group without addition of the inhibitor, indicating that HOP intervention on RCECs may induce apoptosis through activating the JNK signaling pathway.

In summary, HOP intervention of RCECs could increase the intracellular expression of ROS, and ROS increase could regulate JNK inflammatory signaling pathway and CD95/CD95L-mediated cell apoptosis signaling pathway by activating the JNK signaling pathway.

## Materials and Methods

### Ethics Statement

All animal procedures and experiments were approved by the Tongji University School of Medicine Animal Care Committees. Animals were cared for in accordance with the Association of Research for Vision and Ophthalmology statement for the use of Animals in Ophthalmic and Vision Research. All surgery was performed under sodium pentobarbital anesthesia, and all efforts were made to minimize suffering.

### Cell culture

New Zealand Rabbits (Tongji University Laboratorial Animal Center) weigh 2–2.5 kg. The culture of RCECs was performed as described previously [31]. Briefly, RCECs were cultured in Keratinocyte-SFM (1∶1) medium (Gibco) containing 5% fetal bovine serum (FBS), 0.05 mg/ml bovine pituitary extract, 5 ng/ml epidermal growth factor, 100 U/ml penicillin and 100 µg/ml streptomycin. All cells were cultured on the culture dishes, except for cells used for identification; they were cultured on smear at a cell density of 1*10^4^ cells/ml. The third to fourth passage epithelial cells were used in the following experiments, including identification with cytokeratin3/2p monoclonal antibody (Santa Cruz Biotechnology, USA). Cells of 80% confluence were washed three times with PBS and switched to a serum-free Keratinocyte-SFM medium containing 100 U/ml penicillin and 100 µg/ml streptomycin overnight. All experiments were performed at least three times on each of three separate sets of cultures that were initiated from different animal corneas.

### Detection of ROS levels in RCECs

Intracellular ROS levels in RCECs were detected using DCFH-DA (Invitrogen, Carlsbad, CA). RCECs were cultured for an additional 24 h in serum-free media with a different osmolarity, 312 or 500 mOsM which was achieved by adding 0 or 90 mM sodium chloride (NaCl), with or without N-Acetyl-L-cysteine (antioxidant and free radical scavenger, NAC) (Sigma-Aldrich, St. Louis, MO, 10 mM), or diphenyliodonium (NADPH oxidase inhibitor, DPI) (Sigma-Aldrich, 10 µM), which were pre-added 40 min before adding NaCl. The osmolarity of the culture media was measured by a vapour pressure osmometer (Knauer GmbH, Germany). After 24 h, confluent RCECs in 6-well plates were collected, centrifuged, washed with PBS, and incubated with 10 µM DCFH-DA at 37°C for 30 min. RCECs incubated with PBS and dimethyl sulfoxide served as negative controls. Fluorescence at 530 nm was measured by flow cytometry (BD FACSCanto™II) and was assumed to be proportional to the concentration of ROS in the cells.

### Western blot

Cells were incubated with the basal medium (normal osmotic pressure, 312mOsM), the basal medium plus 90 mM NaCl (high osmotic pressure, 500mOsM), and the basal medium plus 90 mM NaCl and 10mM NAC, 10 µM DPI or 25 µM JNK inhibitor SP600125 (Sigma-Aldrich), which were pre-added 40 min before adding NaCl. For Western blot to detect phospho-JNK, the cultures were treated with the conditions for a shorter time of 30 minutes. For Western blot to detect Fas Ligand and CD95, the cultures were treated with the conditions as above for 24h. Cells were sonicated in TBS containing protease inhibitors. The supernatants were collected after centrifugation. Proteins of the equal concentration (80 ug lane^−1^) were separated on 10% SDS-PAGE and transferred to PVDF Transfer Membrane (Millipore Corporation, Temecula, CA). The membranes were blocked in TBS containing 0.1% Tween-20 and 5% nonfat dry milk for 2 h, followed by an overnight incubation at 4°C with phospho-SAPK/JNK antibody, SAPK/JNK rabbit mAb (Cell Signaling Technology, Beverly, MA) at 1∶1000 dilutions, and polyclonal to Fas Ligand (Abcam) 1∶200 dilutions or polyclonal to CD95(Abcam) at 1∶1000 dilutions. After rinse in TBST, the membranes were incubated for 2 h with a horseradish peroxidaseconjugated secondary antibody against rabbit IgG (Dako, Glostrup, Denmark) in a 1∶1000 dilution and rinsed with TBST, followed by SuperSignal West Pico Chemiluminent Substrates (PIERCE) for detecting the blots. The densities of the bands were analyzed by Gel-Pro Analyzer. The expression of GAPDH (Sigma-Aldrich) was used as an internal control.

### Enzyme linked immunosorbent assay (ELISA)

Cell phospho-SAPK/JNK and phospho-NF-κB concentrations were measured by phospho-SAPK/JNK sandwich Elisa kit (Cell Signaling Technology) and phospho-NF-κB p65 sandwich Elisa kit (Cell Signaling Technology) according to the manufacturer's instructions. The cultures were treated with the same conditions as above for 30 minutes.

Cell supernatant IL-1β concentrations was measured by IL-1β Immunoassay (R & D Systems, Minneapolis, MN) according to the manufacturer's instructions. Cells were incubated for 24 h with the basal medium (normal osmotic pressure, 312mOsM), the basal medium plus 90 mM NaCl (high osmotic pressure, 500mOsM), and the basal medium plus 90mM NaCl and 10mM NAC, 10 µM DPI, 25 µM SP600125 or 5 µM NF-kB inhibitor Bay11-7082(Sigma-Aldrich), which were added 40 minutes before adding the NaCl.

The optical density (OD) was measured at 450 nm on a microtitre plate reader. All measurements were performed in duplicate, and the mean values were computed.

### Quantitative real-time PCR

The cultures were treated with the same conditions as IL-1β concentrations detection. After removing the culture medium, cells were washed with PBS, and then combined with the TRIzol reagent (Invitrogen). Extracted RNA was then quantitated spectrophotometrically at 260 nm, and integrity was assessed by agarose-formaldehyde gel electrophoresis. Total RNA samples were treated with DNase I (RQ1; Promega) and then reverse transcribed using a ReverTra Ace RT–PCR kit (Toyobo, Osaka, Japan) according to the manufacturer's instructions. Primers were designed using DNA Star software according to the guidelines supplied with the software. TNF-α primer sequences are 5′-CGTGGCGGCCGACAGAAACT-3′, 5′- AGGAGCACGTAGGAGCGGCA -3′. GAPDH primer sequences are 5′- CCGCCGATGCCCCCATGTTT -3′, 5′- CGACCGACACGTTGGGGGTG -3′.

### Cell apoptosis by TUNEL and Hoechst33342 fluorescent staining assay

TdT-mediated dUTP nick end labeling and Hoechst33342 apoptosis fluorescent staining were used to detect morphological changes of cell apoptosis according to the manufacturer's instructions. Cells were incubated with the basal medium (normal osmotic pressure, 312mOsM), the basal medium plus 90 mM NaCl (high osmotic pressure, 500mOsM), and the basal medium plus 90 mM NaCl and 10 mM NAC, or 10 µM DPI, 25 µM SP600125, which were added 40 minutes before adding the NaCl. All samples were directly analyzed under fluorescence microscope.

### Flow cytometry analysis of apoptosis

The cultures were treated with the same conditions as above. RCECs were stained with FITC-conjugated annexin V and propidium iodide (PI). RCECs were trypsinized and collected for the detection of apoptosis by using Annexin V-FITC Apoptosis Detection Kit (eBioscience, San Diego, CA, USA) according to the manufacturer's instructions. Briefly, RCECs were washed twice with cold PBS and resuspended in 500 µl binding buffer at a concentration of 1 × 10^6^ cells/ml. After the addition of 5 µl Annexin V-FITC solution and 5 µl PI, the cells were incubated for 15 min at room temperature in the dark and then analyzed by flow cytometer (BD FACSCanto™?).

### Statistical analysis

All results were expressed as mean ± standard deviation (SD) unless indicated otherwise notified. Statistical evaluation was performed with the statistics programmer SPSS 14.0 for Windows using ANOVA with multiple comparisons between groups and Pearson correlation tests. Statistical significance was accepted for *P*<0.05.

## Supporting Information

Figure S1 **Melting curve and amplification plots of TNF-α real-time PCR experiments.** TNF-α and GAPDH PCR Amp/Cycle and melt curve peak chart.
(TIF)
