# Supplementary figures and images for: Effect of Reactive Oxygen Species Generation in Rabbit Corneal Epithelial Cells on Inflammatory and Apoptotic Signaling Pathways in the Presence of High Osmotic Pressure
Source: PLoS One. 2013 Aug 15;8(8):e72900. doi: 10.1371/journal.pone.0072900 (PMC3744495; doi:10.1371/journal.pone.0072900)

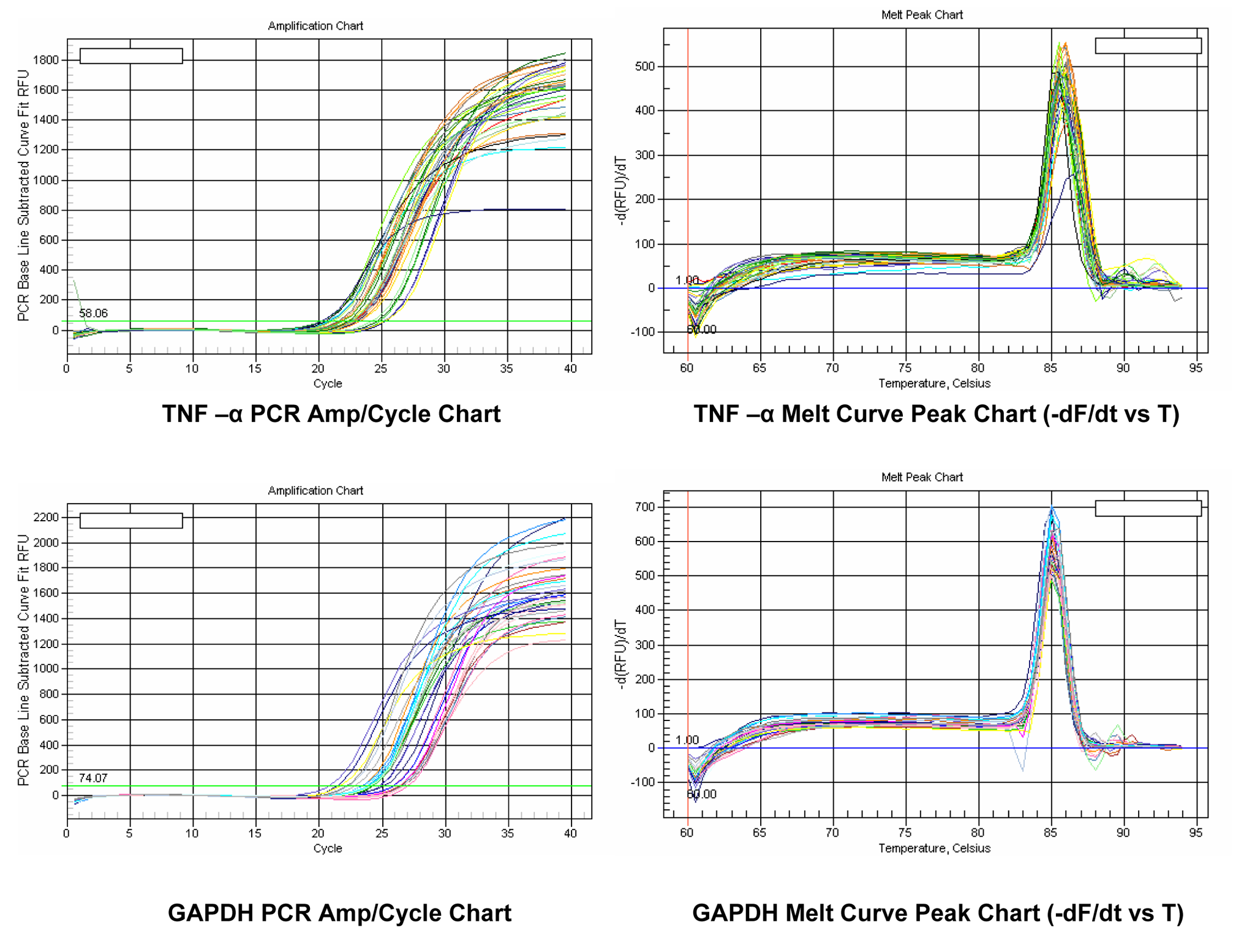

Supplement: Figure S1 — Melting curve and amplification plots of TNF-α real-time PCR experiments. TNF-α and GAPDH PCR Amp/Cycle and melt curve peak chart. (TIF) [file pone.0072900.s001.tif]
